# Sensitivity of Diffusion Tensor Imaging for Assessing Injury Severity in a Rat Model of Isolated Diffuse Axonal Injury: Comparison with Histology and Neurological Assessment

**DOI:** 10.3390/ijms26157333

**Published:** 2025-07-29

**Authors:** Vladislav Zvenigorodsky, Benjamin F. Gruenbaum, Ilan Shelef, Dmitry Frank, Beatris Tsafarov, Shahar Negev, Vladimir Zeldetz, Abed N. Azab, Matthew Boyko, Alexander Zlotnik

**Affiliations:** 1 Department of Radiology, Soroka University Medical Center and the Faculty of Health Sciences, Ben-Gurion University of the Negev, Beer-Sheva 84101, Israel; zvenigorodsky@gmail.com (V.Z.); shelef@bgu.ac.il (I.S.); 2 Department of Anesthesiology and Perioperative Medicine, Mayo Clinic, Jacksonville, FL 32224, USA; gruenbaum.benjamin@mayo.edu; 3 Department of Anesthesiology and Critical Care, Soroka University Medical Center, Ben-Gurion of the Negev, Beer-Sheva 84101, Israel; frdima16@gmail.com (D.F.); shahar.shoshani@gmail.com (S.N.); zlotnika@bgu.ac.il (A.Z.); 4 Department of Histology, Soroka University Medical Center and the Faculty of Health Sciences, Ben-Gurion University of the Negev, Beer-Sheva 84101, Israel; beatriceza@clalit.org.il; 5 Department of Emergency Medicine, Soroka University Medical Center, Ben-Gurion University of the Negev, Beer-Sheva 84101, Israel; zalds@bgu.ac.il; 6 Department of Nursing, Recanati School for Community Health Professions, Faculty of Health Sciences, Ben-Gurion University of the Negev, Beer-Sheva 84101, Israel; azab@bgu.ac.il

**Keywords:** axonal injury, diffusion tensor imaging, histology, magnetic resonance imaging, neurological assessment, traumatic brain injury

## Abstract

Diffuse axonal brain injury (DAI) is a common, debilitating consequence of traumatic brain injury, yet its detection and severity grading remain challenging in clinical and experimental settings. This study evaluated the sensitivity of diffusion tensor imaging (DTI), histology, and neurological severity scoring (NSS) in assessing injury severity in a rat model of isolated DAI. A rotational injury model induced mild, moderate, or severe DAI in male and female rats. Neurological deficits were assessed 48 h after injury via NSS. Magnetic resonance imaging, including DTI metrics, such as fractional anisotropy (FA), relative anisotropy (RA), axial diffusivity (AD), mean diffusivity (MD), and radial diffusivity (RD), was performed prior to tissue collection. Histological analysis used beta amyloid precursor protein immunohistochemistry. Sensitivity and variability of each method were compared across brain regions and the whole brain. Histology was the most sensitive method, requiring very small groups to detect differences. Anisotropy-based MRI metrics, especially whole-brain FA and RA, showed strong correlations with histology and NSS and demonstrated high sensitivity with low variability. NSS identified injury but required larger group sizes. Diffusivity-based MRI metrics, particularly RD, were less sensitive and more variable. Whole-brain FA and RA were the most sensitive MRI measures of DAI severity and were comparable to histology in moderate and severe groups. These findings support combining NSS and anisotropy-based DTI for non-terminal DAI assessment in preclinical studies.

## 1. Introduction

Traumatic brain injury (TBI) remains a leading cause of death and long-term disability worldwide, contributing to significant socioeconomic burden due to prolonged hospitalization, rehabilitation needs, and cognitive and emotional impairments [1,2]. Neuropsychiatric complications, including cognitive deficits, changes in social behavior, depression, and anxiety, are among the most prevalent and disabling outcomes of TBI, affecting up to 50–60% of patients during recovery [3]. These symptoms often persist well beyond the initial trauma, and in many cases do not fully resolve [4]. This underscores the need for robust, sensitive experimental models and assessment methods to investigate the underlying pathophysiology and guide therapeutic development.

Animal models, particularly with rodents, play a critical role in TBI research, offering controlled experimental conditions and the ability to track structural, functional, and behavioral changes over time. Histological techniques remain the gold standard for assessing neuropathological damage, providing cellular-level detail and high specificity [5,6]. However, because these methods are terminal, they preclude longitudinal follow-up and often require large cohorts to track changes across timepoints [6].

Magnetic resonance imaging (MRI), and, particularly, diffusion tensor imaging (DTI), has emerged as a valuable non-invasive modality for investigating TBI in vivo [7]. MRI provides detailed anatomical and functional data, while DTI quantifies white matter integrity, allowing detection of microstructural changes that characterize diffuse axonal brain injury (DAI) [8,9]. Unlike focal injuries, DAI may not appear on conventional imaging but is associated with substantial cognitive and behavioral dysfunction [10,11]. It is a defining feature of many TBI cases and likely contributes significantly to long-term consequences [12]. In addition, the severity of DAI appears to correlate with poorer recovery [13,14].

Like MRI, DTI is an essential tool to study central nervous system (CNS) disorders. Its ability to assess white matter integrity is applied for conditions including multiple sclerosis, Alzheimer’s disease, Parkinson’s disease, epilepsy, stroke, TBI and other brain and spinal cord injuries [15,16]. In addition, it may be useful as a resource to identify functional decline in aging [17]. DTI has also proven valuable in evaluating peripheral nervous system pathologies. In particular, high-field DTI has been successfully utilized in analyzing acute traumatic peripheral nerve injury [18], to evaluate morphometric and microstructural changes in median, tibial, and sural nerves in patients with type 2 diabetes [19], and to predict outcomes in patients with traumatic injuries of the median nerve [20]. These broad applications underscore the versatility of DTI as a quantitative imaging modality for investigating both central and peripheral nervous system injuries and situate it as a critical aid in neurological research.

In our previous studies, we established a rat model of isolated DAI that replicates axonal damage without associated cortical contusion [3,8,21]. This model allowed us to isolate the effects of axonal injury, revealing a strong association between DAI and enduring depression- and anxiety-like behaviors [22]. These results suggest that behavioral symptoms commonly observed in mixed models of TBI may primarily arise from axonal damage. However, few studies have systematically evaluated how histological, neuroimaging, and behavioral measures compare in their ability to characterize the severity of DAI.

Despite the widely acknowledged usefulness of DTI, histology, and behavioral assessments in preclinical TBI models, there are few studies that apply these models holistically to DAI in vivo. Prior research on the topic either uses a single assessment or has not adequately quantified a comparative approach featuring each method across DAI severities. Our study fills this research gap by novelly comparing DTI, histological markers of axonal injury, and neurological severity scores within the same cohort of rats subjected to mild, moderate, or severe DAI. Additionally, we seek to determine which DTI parameters and brain regions of interest (ROIs) are most informative for characterizing injury severity in this rat model. We quantify the minimum group sizes necessary to identify significance between the models and we determine which DTI metrics and brain regions are the most sensitive to axonal injury. Thus, we provide a practical structure for an optimal preclinical study design that accounts for the benefits and limitations of each assessment tool.

This comprehensive approach establishes evidence-based recommendations for DAI monitoring in experimental monitoring, with critical implications for basic research and translational applications in the research of TBI and its secondary conditions.

## 2. Results

### 2.1. Neurological Severity Score (NSS)

The NSS for each group 48 h after DAI appears in Table 1. As hypothesized, we found no difference between groups at baseline before intervention. The sham-operated group did not show any neurological deficit at 48 h after DAI (NSS-0). The NSS at 48 h was significantly greater after mild (1(0–3) vs. 0(0–0.25), U = 74, *p* < 0.05, r = 0.41), moderate (2(1–3) vs. 0(0–0.25), U = 30, *p* < 0.01, r = 0.69), and severe (4.5(3–7.5) vs. 0(0–0.25), U = 30, *p* < 0.01, r = 0.69) DAI, according to the Mann–Whitney U test. The data are measured as a count and expressed as median and 25–75 percentile range. No significant difference was observed between males and females.

### 2.2. Analysis of Immunohistochemistry for β-Amyloid Precursor Protein (β-APP)

The data were analyzed with a two-way ANOVA to determine the effect of the brain trauma (DAI or sham) and sex (male or female).

#### 2.2.1. β-APP Accumulation in the Thalamus

The results of the two-way ANOVA revealed a significant main effect of the brain trauma groups on β-APP accumulation in the thalamus (F(3,56) = 39.74, *p* < 0.001, η^2^ = 0.680), indicating that different levels of diffuse DAI were associated with significantly different levels of β-APP expression. In contrast, the main effect of sex was not significant (F(1,56) = 0.057, *p* = 0.813, η^2^ = 0.001), and there was no significant interaction between sex and brain trauma group (F(3,56) = 0.005, *p* = 0.999, η^2^ = 0.000). Because neither sex nor the interaction term had a significant effect, male and female data were pooled to increase statistical power in subsequent analyses.

**Figure 1:**
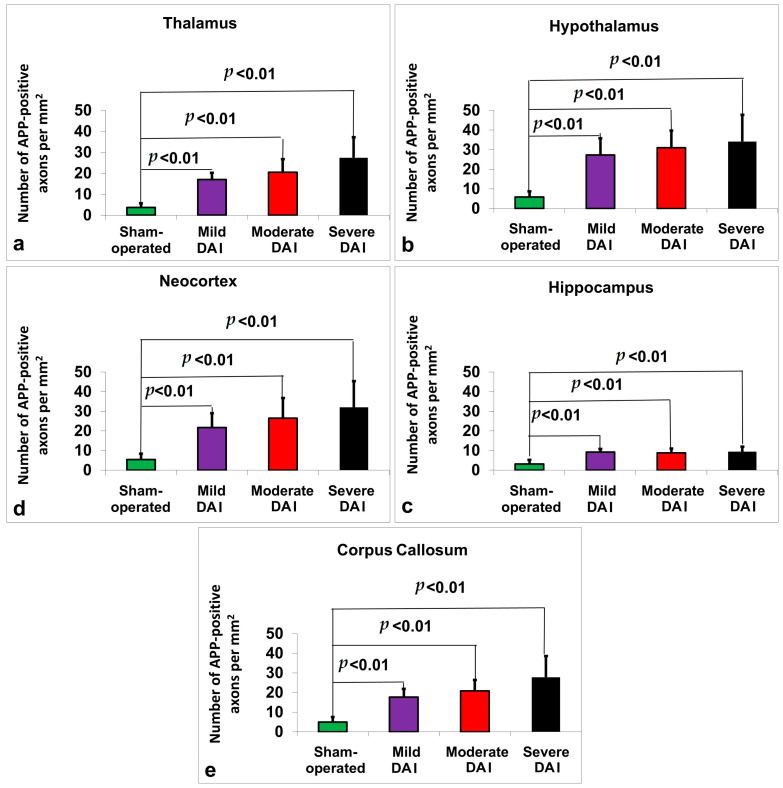
Quantification of β-APP-positive axonal profiles across different brain regions in sham-operated, mild, moderate, and severe DAI groups. Graphs show the number of β-APP-positive axons per mm^2^ in the (**a**) thalamus, (**b**) hypothalamus, (**c**) hippocampus, (**d**) neocortex, and (**e**) corpus callosum. All DAI groups exhibited significantly increased β-APP staining compared to the sham group (*p* < 0.01). Axonal pathology increased with injury severity across all regions. Bars represent mean ± standard deviation.

Post hoc pairwise comparisons with Bonferroni correction showed that all injury groups (mild, moderate, and severe DAI) had significantly higher β-APP levels compared to the sham-operated group (*p* < 0.001 in all comparisons, see Figure 1a). Specifically, mean β-APP levels were 3.75 ± 1.91 in the sham group, 17.06 ± 3.17 in the mild DAI group, 20.50 ± 6.29 in the moderate DAI group, and 27.44 ± 9.77 in the severe DAI group. Significant differences were also found between the mild and severe DAI groups (*p* < 0.001), and between the moderate and severe groups (*p* = 0.018), while no significant difference was observed between the mild and moderate groups (*p* = 0.774).

#### 2.2.2. β-APP Accumulation in the Hypothalamus

The results of the two-way ANOVA revealed a significant main effect of the brain trauma groups on β-APP accumulation in the hypothalamus (F(3,56) = 28.46, *p* < 0.001, η^2^ = 0.604), indicating that different levels of diffuse DAI were associated with significantly different levels of β-APP expression. In contrast, the main effect of sex was not significant (F(1,56) = 0.006, *p* = 0.938, η^2^ = 0.000), and there was no significant interaction between Gender and Study Group (F(3,56) = 0.010, *p* = 0.999, η^2^ = 0.001). Because neither sex nor the interaction term had a significant effect, male and female data were pooled to increase statistical power in subsequent analyses.

Post hoc pairwise comparisons with Bonferroni correction showed that all injury groups (mild, moderate, and severe DAI) had significantly higher β-APP levels compared to the sham-operated group (*p* < 0.001 in all comparisons, see Figure 1b). Specifically, mean β-APP levels were 5.88 ± 2.87 in the sham group, 27.31 ± 8.47 in the mild DAI group, 30.94 ± 8.81 in the moderate DAI group, and 34.13 ± 13.59 in the severe DAI group. Additionally, a significant difference was observed between the sham and mild groups (*p* < 0.001), the sham and moderate groups (*p* < 0.001), and the sham and severe groups (*p* < 0.001). Furthermore, the severe DAI group showed significantly higher β-APP levels than the mild group (*p* = 0.001). No significant differences were found between the mild and moderate groups, or between the moderate and severe groups.

#### 2.2.3. β-APP Accumulation in the Hippocampus

The results of the two-way ANOVA revealed a significant main effect of the brain trauma groups on β-APP accumulation in the hippocampus (F(3,56) = 28.98, *p* < 0.001, η^2^ = 0.608), indicating that different levels of diffuse DAI were associated with significantly different levels of β-APP expression. In contrast, the main effect of sex was not significant (F(1,56) = 0.051, *p* = 0.823, η^2^ = 0.001), and there was no significant interaction between sex and brain trauma group (F(3,56) = 0.169, *p* = 0.917, η^2^ = 0.009). Because neither sex nor the interaction term had a significant effect, male and female data were pooled to increase statistical power in subsequent analyses.

Post hoc pairwise comparisons with Bonferroni correction showed that all injury groups (mild, moderate, and severe DAI) had significantly higher β-APP levels in the hippocampus compared to the sham-operated group (*p* < 0.001 in all comparisons). Specifically, mean β-APP levels were 3.19 ± 2.10 in the sham group, 9.19 ± 1.60 in the mild DAI group, 8.81 ± 2.20 in the moderate DAI group, and 9.44 ± 2.61 in the severe DAI group. A significant difference was observed between the sham and mild groups (*p* < 0.001), the sham and moderate groups (*p* < 0.001), and the sham and severe groups (*p* < 0.001). No significant differences were found between the mild and moderate groups, the mild and severe groups, or the moderate and severe groups. See Figure 1c.

#### 2.2.4. β-APP Accumulation in the Neocortex

The results of the two-way ANOVA revealed a significant main effect of the brain trauma groups on β-APP accumulation in the neocortex (F(3,56) = 22.13, *p* < 0.001, η^2^ = 0.542), indicating that different levels of diffuse DAI were associated with significantly different levels of β-APP expression. In contrast, the main effect of sex was not significant (F(1,56) = 0.008, *p* = 0.928, η^2^ = 0.0001), and there was no significant interaction between sex and brain trauma groups F(3,56) = 0.052, *p* = 0.984, η^2^ = 0.003). Because neither sex nor the interaction term had a significant effect, male and female data were pooled to increase statistical power in subsequent analyses.

Post hoc pairwise comparisons with Bonferroni correction showed that all injury groups (mild, moderate, and severe DAI) had significantly higher β-APP levels compared to the sham-operated group (*p* < 0.001 in all comparisons). Specifically, mean β-APP levels were 5.38 ± 3.05 in the sham group, 21.75 ± 7.25 in the mild DAI group, 25.69 ± 10.43 in the moderate DAI group, and 32.00 ± 13.38 in the severe DAI group. A significant difference was observed between the sham and mild groups (*p* < 0.001), the sham and moderate groups (*p* < 0.001), and the sham and severe groups (*p* < 0.001). Additionally, the severe DAI group showed significantly higher β-APP levels than the mild group (*p* = 0.024). No significant differences were found between the mild and moderate groups, or between the moderate and severe groups. See Figure 1d.

#### 2.2.5. β-APP Accumulation in the Corpus Callosum

A two-way ANOVA was conducted to examine the effects of brain trauma group (sham-operated, mild DAI, moderate DAI, severe DAI) and Gender (female, male) on β-APP immunoreactivity in the corpus callosum.

There was a significant main effect of the brain trauma group on β-APP accumulation, F(3,56) = 34.35, *p* < 0.001, partial η^2^ = 0.648, indicating that the severity of DAI was associated with increasing β-APP burden. There was no significant main effect of sex, F(1,56) = 0.81, *p* = 0.373, partial η^2^ = 0.014, and no significant sex × brain trauma group interaction, F(3,56) = 1.18, *p* = 0.324, partial η^2^ = 0.060, suggesting that the pattern of increasing β-APP immunoreactivity across DAI severity was similar in males and females. Male and female data were pooled to increase statistical power in subsequent analyses.

Post hoc pairwise comparisons with Bonferroni correction showed that all injury groups (mild, moderate, and severe DAI) had significantly higher β-APP levels in the corpus callosum compared to the sham-operated group (*p* < 0.001 in all comparisons). Specifically, mean β-APP levels were 4.94 ± 2.59 in the sham group, 17.69 ± 4.19 in the mild DAI group, 20.88 ± 5.60 in the moderate DAI group, and 27.81 ± 10.78 in the severe DAI group. A significant difference was observed between the sham and mild groups (*p* < 0.001), the sham and moderate groups (*p* < 0.001), and the sham and severe groups (*p* < 0.001). Additionally, β-APP levels were significantly higher in the severe group compared to both the mild (*p* = 0.001) and moderate (*p* = 0.023) groups. No significant difference was found between the mild and moderate groups. See Figure 1e.

### 2.3. MRI-Based Neuroimaging Outcomes

Pairwise Bonferroni-adjusted comparisons and descriptive statistics are summarized in Table 2.

#### 2.3.1. Fractional Anisotropy (FA)

**Figure 2:**
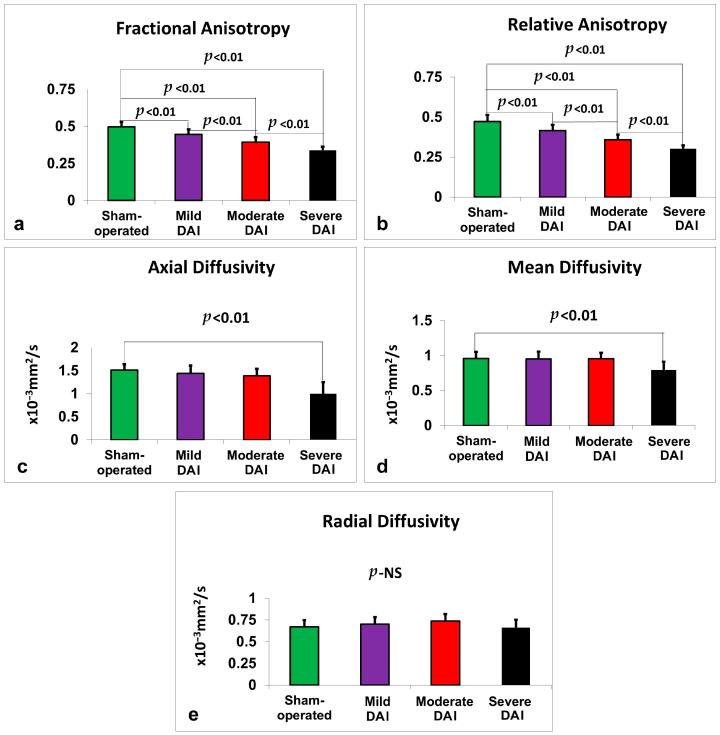
Diffusion MRI metrics measured 48 h after DAI across injury severity groups compared to sham-operated controls. (**a**) FA, (**b**) RA, (**c**) AD, (**d**) MD, and (**e**) RD were quantified from whole-brain regions of interest. FA, RA, AD, and MD values significantly declined with increasing injury severity. No significant group differences were observed for RD. Bars represent mean ± standard error. NS = not significant.

See Figure 2a. In the thalamus, a two-way ANOVA revealed a significant main effect of study group on FA values, F(3,56) = 12.09, *p* < 0.001, partial η^2^ = 0.393, with FA decreasing in association with greater DAI severity. There was no significant effect of gender (*p* = 0.927) or group × gender interaction (*p* = 0.794), so data were pooled across sexes. Similar findings were observed in the hypothalamus, where group differences were significant, F(3,56) = 9.31, *p* < 0.001, partial η^2^ = 0.333, with no gender or interaction effects. In the hippocampus, FA significantly varied by study group, F(3,56) = 11.59, *p* < 0.001, partial η^2^ = 0.383, again with no significant gender effects. The corpus callosum also showed significant group differences, F(3,56) = 7.31, *p* < 0.001, partial η^2^ = 0.281. In the neocortex, FA differences were even more pronounced, F(3,56) = 19.70, *p* < 0.001, partial η^2^ = 0.513, with no significant contributions from gender. Whole-brain FA analysis revealed the strongest group effect, F(3,56) = 72.63, *p* < 0.001, partial η^2^ = 0.796. Across all FA analyses, there were no significant sex or interaction effects, and data were therefore pooled.

#### 2.3.2. Relative Anisotropy (RA)

RA values also declined progressively with increasing injury severity across multiple brain regions (see Figure 2b). In the thalamus, the group effect was significant, F(3,56) = 12.98, *p* < 0.001, partial η^2^ = 0.410. The hypothalamus showed a similar pattern, F(3,56) = 9.15, *p* < 0.001, partial η^2^ = 0.329, as did the hippocampus, F(3,56) = 8.32, *p* < 0.001, partial η^2^ = 0.308. In the corpus callosum, RA differed significantly by group, F(3,56) = 6.61, *p* = 0.001, partial η^2^ = 0.261, and the neocortex demonstrated the largest effect, F(3,56) = 24.26, *p* < 0.001, partial η^2^ = 0.565. Whole-brain RA values similarly declined with severity, F(3,56) = 70.34, *p* < 0.001, partial η^2^ = 0.790. No sex or interaction effects were observed in any region.

#### 2.3.3. Axial Diffusivity (AD)

In the thalamus, AD significantly differed by injury group, F(3,56) = 8.25, *p* < 0.001, partial η^2^ = 0.306, with no effect from sex or interaction (see Figure 2c). Similar findings were seen in the hypothalamus, F(3,56) = 7.95, *p* < 0.001, partial η^2^ = 0.299, and the hippocampus, F(3,56) = 4.91, *p* = 0.004, partial η^2^ = 0.208. The corpus callosum also showed group differences, F(3,56) = 5.53, *p* = 0.002, partial η^2^ = 0.228, as did the neocortex, F(3,56) = 4.67, *p* = 0.006, partial η^2^ = 0.200. In the whole brain, AD strongly decreased with increasing DAI severity, F(3,56) = 24.12, *p* < 0.001, partial η^2^ = 0.564. No effects from sex were found in any region.

#### 2.3.4. Mean Diffusivity (MD)

Significant group effects on MD were found in the thalamus, F(3,56) = 7.80, *p* < 0.001, partial η^2^ = 0.295, hypothalamus, F(3,56) = 5.66, *p* = 0.002, partial η^2^ = 0.233, and corpus callosum, F(3,56) = 9.78, *p* < 0.001, partial η^2^ = 0.344 (see Figure 2d). Whole-brain MD also differed significantly by group, F(3,56) = 9.33, *p* < 0.001, partial η^2^ = 0.333. MD changes in the hippocampus were not significant (*p* = 0.055), nor were those in the neocortex (*p* = 0.385). No sex or interaction effects were observed.

#### 2.3.5. Radial Diffusivity (RD)

In the thalamus, RD differed significantly by injury group, F(3,56) = 4.10, *p* = 0.011, partial η^2^ = 0.180, though post hoc comparisons were not significant (see Figure 2e). The hypothalamus showed a similar pattern, F(3,56) = 2.88, *p* = 0.044, partial η^2^ = 0.134. No significant differences were observed in the hippocampus or neocortex. In the corpus callosum, RD showed a significant group effect, F(3,56) = 8.54, *p* < 0.001, partial η^2^ = 0.314, and a significant group × gender interaction, F(3,56) = 2.85, *p* = 0.045, partial η^2^ = 0.132. This interaction prompted stratified post hoc analysis by sex, results of which are reported in Table 2. At the whole-brain level, RD showed a significant group effect, F(3,56) = 2.80, *p* = 0.048, partial η^2^ = 0.130, but post hoc comparisons were not significant. No other sex-related effects were detected.

### 2.4. Comparison of Sensitivity and Correlation Between NSS, MRI, and Histological Outcomes in a Rat Model of DAI

Correlations between the different measures of DAI severity, including NSS, histology, and MRI determined metrics, were calculated and are shown in Table 3. Required sample sizes for detecting significant differences across NSS, MRI, and histological measures are provided in Table 4, with color highlights in Supplementary Table S1 (raw data) to aid interpretation where needed. A schematic summary comparing each assessment modality can be found in Table 5.

## 3. Discussion

In this study, we evaluated the sensitivity of MRI, histology, and NSS in quantifying injury severity in a rat model of DAI. We aimed to identify the most informative DTI parameters and brain ROIs for assessing DAI using imaging and compared these modalities with the histological gold standard.

We first assessed neurological function 48 h after injury. Motor and behavioral impairments were measured using a modified NSS protocol, which is widely applied in rodent models of closed-head and unilateral TBI [23]. The NSS method used in our lab has been previously validated as a sensitive tool for detecting functional deficits, including in rats with mild TBI compared to sham-operated controls [21]. Our previous findings also indicated that NSS may be more sensitive than some imaging or histological markers in certain contexts [8,21].

In this study, we adapted the NSS for use in the DAI model and identified specific subtests most reflective of diffuse axonal injury. NSS scores showed a clear correlation with injury severity and were able to detect even mild DAI. However, sensitivity was lower than histological analysis, as indicated by higher sample sizes required to detect significant differences across groups (five to fifteen animals for NSS versus only two to three for histology, depending on injury severity). This might be due to the fact that while NSS is a validated and sensitive tool for detecting functional deficits (including in mild TBI) it reflects the behavioral consequences of injury, which can be influenced by compensatory mechanisms, behavioral variability, and non-neural factors [24].

Histological analysis focused on five brain ROIs (thalamus, hypothalamus, neocortex, hippocampus, and corpus callosum) and utilized β-APP immunohistochemistry, a widely accepted method for detecting diffuse axonal injury [25,26]. β-APP accumulates in injured axons due to impaired axoplasmic transport and allows for early and highly sensitive detection of axonal damage. This method is considered the gold standard for identifying DAI in both clinical and experimental settings and has been shown to outperform routine histology, especially in mild cases [27,28]. Consistent with these findings, our results demonstrated that β-APP immunostaining accurately detected mild, moderate, and severe DAI across all examined brain regions. Estimated sample sizes for detecting group differences based on these histological findings were as low as two to three rats, regardless of severity or ROI, further highlighting the method’s sensitivity.

We then compared these findings with MRI results collected prior to tissue harvesting. MRI, particularly DTI, is an essential tool for non-invasive assessment of white matter integrity in DAI. Unlike histology, MRI enables longitudinal evaluation of microstructural changes, making it especially valuable for studies involving behavioral outcomes or therapeutic interventions. In our study, we assessed several DTI-derived parameters, including FA, RA, AD, MD, and RD, across the same five brain ROIs and the entire brain.

Our findings show that anisotropy measures (FA and RA) were more sensitive and consistent than diffusivity measures (AD, MD, RD) for detecting injury. Anisotropy metrics revealed significant changes in all brain regions for moderate and severe injury groups and in four out of six regions for the mild group. In contrast, AD and MD detected significant changes only in severe cases, and RD failed to show significant differences in most comparisons, highlighting its limited utility in this model. Notably, whole-brain FA and RA provided the most robust signal with the least variability across groups. This was especially evident when comparing sample size estimates: FA and RA in the whole brain required only two rats per group to detect differences in moderate and severe injury, similar to histological methods, but required six to eleven rats in mild injury, indicating slightly lower sensitivity in less severe cases.

The reduced variability and higher sensitivity of whole-brain measurements likely reflect both anatomical and technical factors. Specifically, measurements from individual ROIs were based on small circular regions (0.1 mm diameter), where signal strength is inherently lower and more difficult to register. In contrast, whole-brain measurements encompass a larger volume, allowing for stronger and more stable signal acquisition. Given the resolution limitations of the 3 Tesla clinical scanner used in this study, larger sampled areas provide more reliable data. Thus, while ROI-level DTI can offer localized insight, whole-brain analysis appears more reproducible and robust at this scanner strength.

Correlation analysis further supported these findings. The strongest correlations were observed between (1) histological results and anisotropy-based DTI parameters, and (2) histological results and NSS scores. Among MRI outcomes, whole-brain FA and RA showed the highest correlations with NSS, reinforcing their potential as sensitive and reliable measures for evaluating DAI severity.

The measurements of FA, RA, MD, and RD have relevance for the axonal injury model in different ways. FA and RA are particularly important for DAI and related TBI due to their ability to quantify the directionality of water diffusion and therefore detect more sensitively the integrity and formation of axonal fibers [29,30]. MD measures the overall magnitude of water diffusion and RD measures its perpendicular qualities of water diffusion; however, they can be affected by mechanisms besides axonal injury, such as demyelination, edema, and inflammation [31].

We propose that the higher sensitivity of FA and RA in our results is attributable to their direct dependence on the coherence and density of intact axonal bundles, which are preferentially disrupted in DAI. Axonal shearing and cytoskeletal breakdown decrease the directional coherence of water movement, causing a profound decrease in anisotropy-based metrics [29,32]. It is possible that the changes in MD and RD are less pronounced at the 48-h time point that we measured because those metrics can be modified by factors such as extracellular water accumulation (vasogenic edema) or demyelination, which may not be developed or noticeable at that time point [33,34]. In addition, RD and MD have been shown to be sensitive to myelin loss and extracellular fluid alterations, which can occur more noticeably in chronic or demyelinating conditions, substantial inflammation, or edema [31,35]. At the 48-h post-injury stage in the DAI model, we found limited demyelination or vasogenic edema according to the histology. Therefore, we believe that the reduced sensitivity of RD and MD is also caused by the relative absence of those mediating processes at that post-injury time point.

This study presents, for the first time, a systematic comparison between DTI, histology, and behavioral assessments for measuring DAI severity in a rodent model. Our results show that anisotropy-based DTI metrics, especially FA and RA, approach the sensitivity of histology, the gold-standard measurement tool for differentiating moderate and severe axonal injury, and offers a critical advantage in its non-terminal, longitudinal assessment. The benefits of a DTI model include reducing animal use and supporting the study of injury progression and recovery. We confirmed that the NSS is less sensitive, especially for mild injuries, and requires larger sample sizes. Our framework of the variability and statistical power of each modality offers practical guidance for experimental design and endpoint selection. Characterizing the specific DTI parameters and brain regions that we identified as most accurate in reflecting injury severity allows for an improved structure for imaging protocols.

## 4. Materials and Methods

The experiments were conducted in accordance with the recommendations of the Declarations of Helsinki and Tokyo and the Guidelines for the Use of Experimental Animals of the European Community. The experiments were approved by the Animal Care Committee of Ben-Gurion University of the Negev, Israel (Board approval codes: BGU-317-03-2024C and IL-11-02-2019C).

### 4.1. Animals

The experiments included 32 male and 32 female Sprague-Dawley rats from Harlan Laboratories in Israel, each weighing between 280 and 320 g. The rats were supplied with Purina Chow and water ad libitum. Their conditions included a 12-h light/12-h dark cycle and a constant temperature of 22 ± 1 °C. All experimental procedures occurred during the dark phase, between 08:00 and 16:00.

### 4.2. Experimental Design

**Figure 3:**
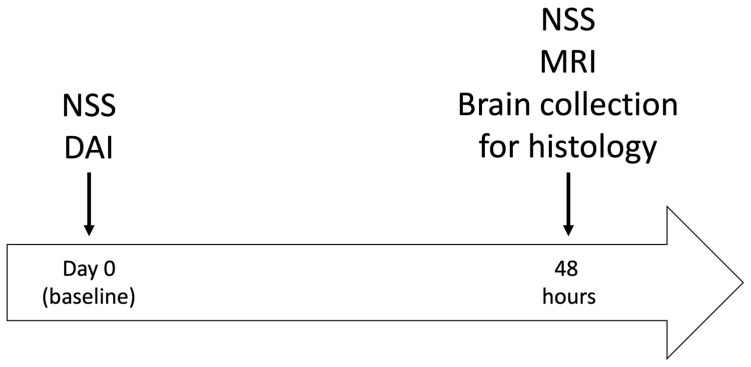
Experimental timeline. NSS—neurological severity score; DAI—diffuse axonal brain injury; MRI—magnetic resonance imaging.

The study was conducted using a 2 × 2 design. The dependent variables were DAI (48 rats), sham (16 rats), and sex (male, 32/female, 32). A total of 64 rats were randomly assigned into one of four groups: sham-operated rats (*n* = 16 rats), mild DAI (*n* = 16), moderate DAI (*n* = 16) and severe DAI (*n* = 16). Neurological severity was ascertained before surgery (baseline) and again at 48 h following various degrees of DAI severity (see Figure 3). After the second neurological evaluation, all rats were scanned on a clinical MRI scanner and subsequently euthanized for histological evaluation.

### 4.3. Neurological Severity Score (NSS)

NSS was independently established by two evaluators blinded to the study groups, based on criteria adapted from established protocols [21,36]. They scored motor and behavioral impairments, with scores ranging from 0 (no deficits) to 15 (maximal impairment) using a series of five tasks: movement on a wide platform (rated on a 3-point scale), movement on a narrow platform (4-point scale), ability to stay on the narrow platform (2-point scale), performance during beam traversal (3-point scale), and balance while not moving on the beam (3-point scale).

### 4.4. Induction of DAI

The apparatus to induce DAI has been previously developed and described by our group [8,36]. It consists of four primary components: (1) a transparent cylindrical chamber; (2) a weighted iron mass; (3) a rotational assembly comprising a cylindrical tube, dual bearings, and a head fixation unit with ear pins; and (4) a horizontal platform supported by two bearings.

Rats were first anesthetized with isoflurane (5% induction, 1.5–2.5% maintenance) delivered in a 1:1 mixture of medical air and oxygen. The animal’s head was then secured in the apparatus using ear pins placed into the external auditory canal on the side of impact. A 2 kg weight was then released from a predetermined height, depending on the injury severity group: 100 cm (mild), 150 cm (moderate), or 180 cm (severe). Upon striking a bolt, the falling weight triggered the rotational mechanism, causing the rat’s head to rotate rapidly from 0° to 90°. Following the procedure, animals were monitored in a recovery chamber.

### 4.5. Histology

In order to measure the density of β-APP-positive axons in the rats’ brains, immunohistochemistry staining was done 48 h after the MRI (approximately 12–24 h post-DAI). The rats’ chests were exposed, and the animals were perfused with cooled saline through the left ventricle at a pressure of 110 mm Hg until colorless perfusion fluid was obtained from the right atrium, followed by 500 mL of 4% paraformaldehyde in 0.1 M phosphate buffer saline (pH 7.4). The brain was removed immediately and fixed in 4% buffered paraformaldehyde solution for 48 h at 4 °C. The brain was then divided into 5 mm coronal slices from the olfactory bulb to the visual cortex. After paraffin embedding, slices of 5 μm were excised using microtome sectioning [36]. Immunochemical staining and examination were performed as detailed previously [36].

### 4.6. Diffusion-Weighted Imaging (DWI)

A 3T MRI (Ingenia, Philips Medical Systems, Best, The Netherlands) with an eight-channel receive-only coil was used to perform DWI at 48 h following intervention, as described previously [21]. The animals were maintained under general anesthesia (1.5% isoflurane in oxygen). Diffusion tensor imaging in six directions was conducted axially using a multi-shot, spin-echo, echo-planar sequence with TR/TE = 1419/138 msec and an epi factor of 19, SENSE factor = 1.5 and a b-factor = 1000 s/mm^2^, and spectrally-selective fat suppression. Seven slices were acquired with zero gap. The resolution (freq × phase × slice) was 0.55 × 0.55 × 2.0 mm. Five signal averages were collected for a scan time of 11:19 min. The Intellispace Portal workstation (V5.0.0.20030, Philips Medical Systems, Best, The Netherlands) was used for the post-processing of the permeability and perfusion studies.

### 4.7. Regions of Interest (ROI)

To enable group-wide analysis, images were transformed into a common anatomical space by constructing a nonlinear composite brain from individual scans. ROIs were then manually delineated on this average brain by aligning it based on the Paxinos and Watson Rat Brain Atlas [37]. ROIs were defined for both left and right thalamus, hypothalamus, hippocampus, corpus callosum and neocortex. DTI metrics were calculated by averaging values from the bregma −3.14 mm image slice across the left and right hemispheres for each region of interest.

### 4.8. Diffusion-Weighted Imaging (DWI) Parameter Map Analysis

An image analysis was conducted by an experienced evaluator who was blinded to group allocations. Fractional anisotropy (FA) and relative anisotropy (RA) maps were generated using the Philips MRI software platform (version 5.7.1.2, Ingenia, Philips Medical Systems, Best, The Netherlands). For each predefined ROI, DTI metrics were calculated and averaged [38]. These averaged values underwent statistical analysis [8,38,39].

### 4.9. Statistical Analysis

Statistical evaluation was carried out using IBM SPSS Statistics for Windows, Version 24.0 (IBM Corp., Armonk, NY, USA). A Kolmogorov–Smirnov test determined the correct test for comparisons between the different parameters. The data were analyzed with two-way ANOVA for the effects of brain trauma and sex. Post hoc Bonferroni tests or planned pair comparisons were performed when the interactions were significant. Using Pearson’s test (for parametric data) and Spearman’s test (for non-parametric data), we calculated the correlation between NSS, histological and MRI outcome. The NSS were compared by the Mann–Whitney U test. Normally distributed data and continuous variables were presented as mean ± SD. Non-parametric data were presented as median ± interquartile range. The results were considered statistically significant when *p* < 0.05.

## 5. Conclusions

This study demonstrates that histology remains the most accurate and sensitive method for detecting DAI severity in a rat model, capable of identifying even mild injuries with minimal group sizes. Among MRI techniques, anisotropy-based DTI (FA and RA) outperformed diffusivity-based metrics and closely approximated histological sensitivity in moderate and severe injuries. Whole-brain anisotropy measures provided the most consistent and least variable results across injury groups, making them the preferred imaging ROI in 3 Tesla applications. NSS remains a useful and accessible tool for assessing functional outcomes, though it is somewhat less sensitive than imaging or histological methods. Together, these findings support the combined use of NSS and whole-brain anisotropy DTI for sensitive, non-terminal evaluation of DAI in longitudinal preclinical studies. Our observations fill a gap in preclinical neurological injury research and support the use of DTI as a translational modality to supplement or replace histological approaches in clinical settings. We anticipate that our study will have long-term implications for therapeutic monitoring, outcome prediction, and severity-based treatment approaches in TBI and related conditions.

## Figures and Tables

**Table 1:** Determination of Neurological Severity Score 48 h after DAI. There was a significant difference in NSS between the DAI group at 48 h after DAI and the sham-operated group, marked with a star (* *p* ≤ 0.05 and ** *p* ≤ 0.01). The data are measured as counts and presented as median and range.

NSS Values of the Study Groups		
Animal Groups	N	Median (Range)
Sham-Operated	16	0(0–0.25)
DAI Mild	16	1(0–3) *
DAI Moderate	16	2(1–3) **
DAI Severe	16	4.5(3–7.5) **

**Table 2:** Diffusion MRI metrics including axial diffusivity (AD), fractional anisotropy (FA), radial diffusivity (RD), relative anisotropy (RA), and mean diffusivity (MD) were measured in six brain regions of interest: thalamus, hypothalamus, hippocampus, corpus callosum, neocortex, and whole brain. Data were collected from four experimental groups: sham operated controls and rats with mild, moderate, or severe diffuse DAI, with equal numbers of males and females in each group (*n* = 16 per each group). Two-way ANOVA was used for each region and metric, with injury group and sex as independent variables. When no significant sex effects or interactions were found, data were pooled and reported as mean ± standard deviation. Where sex differences were present, results are shown separately for males (M) and females (F). Post hoc comparisons were performed when appropriate. Asterisks indicate statistical significance between groups: * *p* ≤ 0.05; ** *p* ≤ 0.01. Variability is presented as the percentage coefficient of variation.

MRI	ROI	Study Groups ($\bar{x}$ ± SD.; Variability; * *p* ≤ 0.05 and ** *p* ≤ 0.01)			
		Sham-Operated	Mild DAI	Moderate DAI	Severe DAI
Fractional Anisotropy	Thalamus	0.49 ± 0.07; 15	0.42 ± 0.14; 34; *	0.33 ± 0.08; 26; **	0.31 ± 0.05; 16; **
	Hypothalamus	0.42 ± 0.06; 16	0.37 ± 0.09; 24	0.33 ± 0.04; 12; **	0.3 ± 0.07; 24; **
	Hippocampus	0.38 ± 0.07; 17	0.34 ± 0.07; 20	0.28 ± 0.06; 21; **	0.25 ± 0.07; 26; **
	Corpus Callosum	0.47 ± 0.12; 25	0.37 ± 0.08; 21; *	0.31 ± 0.07; 21; **	0.42 ± 0.12; 28
	Neocortex	0.43 ± 0.07; 16	0.34 ± 0.08; 23; **	0.26 ± 0.04; 17; **	0.29 ± 0.07; 24; **
	W. Brain	0.5 ± 0.03; 7	0.45 ± 0.03; 7; **	0.39 ± 0.03; 8; **	0.34 ± 0.02; 7; **
	$\bar{x}$ variability between all ROI	16	21	16	21
Relative Anisotropy	Thalamus	0.45 ± 0.08; 17	0.38 ± 0.15; 39	0.27 ± 0.07; 26; **	0.27 ± 0.05; 17; **
	Hypothalamus	0.38 ± 0.07; 18	0.33 ± 0.09; 27	0.28 ± 0.04; 14; **	0.26 ± 0.07; 25; **
	Hippocampus	0.32 ± 0.07; 22	0.29 ± 0.06; 23	0.24 ± 0.06; 27; **	0.21 ± 0.06; 27; **
	Corpus Callosum	0.43 ± 0.13; 29	0.33 ± 0.08; 23; *	0.27 ± 0.06; 24; **	0.39 ± 0.15; 38
	Neocortex	0.4 ± 0.08; 19	0.29 ± 0.07; 25; **	0.22 ± 0.04; 18; **	0.25 ± 0.07; 26; **
	W. Brain	0.47 ± 0.04; 9	0.42 ± 0.04; 9; **	0.36 ± 0.03; 9; **	0.3 ± 0.02; 7; **
	$\bar{x}$ variability between all ROI	19	24	20	24
Axial Diffusivity	Thalamus	1.21 ± 0.21; 18	1.2 ± 0.34; 28	1.08 ± 0.1; 9	0.81 ± 0.3; 37; **
	Hypothalamus	1.51 ± 0.31; 20	1.45 ± 0.47; 32	1.23 ± 0.23; 19	0.99 ± 0.26; 26; **
	Hippocampus	1.33 ± 0.26; 20	1.17 ± 0.17; 15	1.19 ± 0.18; 15	1.05 ± 0.21; 20; **
	Corpus Callosum	1.24 ± 0.35; 29	1.21 ± 0.16; 14	1.1 ± 0.16; 15	0.93 ± 0.22; 23; **
	Neocortex	1.23 ± 0.19; 15	1.04 ± 0.18; 17; *	1.03 ± 0.16; 15; *	1.02 ± 0.2; 19; *
	W. Brain	1.51 ± 0.13; 9	1.44 ± 0.17; 12	1.39 ± 0.15; 11	1 ± 0.25; 26; **
	$\bar{x}$ variability between all ROI	20	20	14	25
Mean Diffusivity	Thalamus	0.83 ± 0.16; 19	0.87 ± 0.15; 17	0.8 ± 0.08; 10	0.61 ± 0.22; 36; **
	Hypothalamus	1.06 ± 0.27; 25	1.05 ± 0.28; 26	1 ± 0.29; 28	0.73 ± 0.15; 21; **
	Hippocampus	0.95 ± 0.17; 17	0.86 ± 0.11; 12	0.91 ± 0.11; 12	0.83 ± 0.13; 16
	Corpus Callosum	0.85 ± 0.13; 16	0.86 ± 0.09; 11	0.84 ± 0.1; 13	0.65 ± 0.17; 26; *
	Neocortex	0.84 ± 0.15; 17	0.77 ± 0.12; 16	0.81 ± 0.11; 14	0.78 ± 0.13; 16
	W. Brain	0.96 ± 0.09; 10	0.95 ± 0.11; 11	0.95 ± 0.09; 9	0.8 ± 0.12; 15; **
	$\bar{x}$ variability between all ROI	17	16	15	22
Radial Diffusivity	Thalamus	0.57 ± 0.12; 21	0.67 ± 0.15; 22	0.66 ± 0.1; 15	0.54 ± 0.14; 25
	Hypothalamus	0.81 ± 0.23; 29	0.86 ± 0.34; 39	0.83 ± 0.23; 28	0.63 ± 0.11; 18
	Hippocampus	0.74 ± 0.13; 17	0.71 ± 0.09; 13	0.77 ± 0.09; 11	0.73 ± 0.1; 14
	Corpus Callosum	F 0.56 ± 0.11; 20 M 0.7 ± 0.09; 14	F 0.7 ± 0.09; 13 M 0.68 ± 0.1; 14	F 0.75 ± 0.11; 14 ** M 0.67 ± 0.06; 9	F 0.54 ± 0.12; 22 M 0.49 ± 0.21; 42 *
	Neocortex	0.64 ± 0.12; 18	0.63 ± 0.11; 17	0.71 ± 0.1; 14	0.66 ± 0.11; 17
	W. Brain	0.67 ± 0.08; 12	0.7 ± 0.08; 11	0.74 ± 0.08; 11	0.66 ± 0.09; 14
	$\bar{x}$ variability between all ROI	19	19	15	20

**Table 3:** Comparison of DAI Severity Outcome Measures. Correlation analysis was performed to assess the relationship between different measures of DAI severity 48 h after injury, including neurological severity score (NSS), histological findings, and MRI-derived metrics. Spearman’s correlation coefficient (rs) was used for non-parametric data, and Pearson’s correlation coefficient (rp) was used for parametric data. Correlations were calculated between NSS, MRI parameters, and histological results. ** Significance is indicated as follows: * *p* ≤ 0.05, ** *p* ≤ 0.01, NS = not significant.

DTI	ROI	Immunohistochemistry for β-APP					
		Thalamus	Hypothalamus	Hippocampus	Corpus Callosum	Neocortex	NSS
Axial Diffusivity	Thalamus	rs = 0.374 **					NS
	Hypothalamus		rp = 0.302 *				rs = 0.373 **
	Hippocampus			rs = 0.257 *			NS
	Corpus Callosum				rs = 0.293 *		rs = 0.336 **
	Neocortex					rs = 0.262 *	NS
	Whole Brain						rs = 0.424 **
Fractional Anisotropy	Thalamus	rp = 0.728 **					rs = 0.632 **
	Hypothalamus		rp = 0.765 **				rs = 0.7 **
	Hippocampus			rp = 0.696 **			rs = 0.719 **
	Corpus Callosum				rs = 0.461 **		rs = 0.445 **
	Neocortex					rs = 0.822 **	rs = 0.646 **
	Whole Brain						rs = 0.727 **
Radial Diffusivity	Thalamus	rp = 0.728 **					NS
	Hypothalamus		NS				rs = 0.259 *
	Hippocampus			NS			NS
	Corpus Callosum				NS		NS
	Neocortex					NS	NS
	Whole Brain						NS
Relative Anisotropy	Thalamus	rs = 0.843 **					rs = 0.655 **
	Hypothalamus		rp = 0.786 **				rs = 0.718 **
	Hippocampus			rp = 0.686 **			rs = 0.785 **
	Corpus Callosum				rs = 0.585 **		rs = 0.529 **
	Neocortex					rs = 0.839 **	rs = 0.69 **
	Whole Brain						rs = 0.769 **
Mean Diffusivity	Thalamus	rs = 0.28 *					rs = 0.264 *
	Hypothalamus		rs = 0.291 *				rs = 0.336 **
	Hippocampus			NS			NS
	Corpus Callosum				NS		NS
	Neocortex					NS	NS
	Whole Brain						rs = 0.251 *
NSS		rs = 0.745 **	rs = 0.795 **	rs = 0.714 **	rs = 0.816 **	rs = 0.772 **	

**Table 4:** Estimated sample sizes for NSS, MRI, and histological outcome measures. Minimum sample sizes per group required to detect statistically significant differences between control and treatment groups are shown for NSS, MRI, and histological outcomes. Estimates are based on observed means and standard deviations, assuming 95% confidence (α = 0.05) and 80% power (β = 0.2). Values reflect the specific effect sizes and variability observed in each modality. For calculated sample sizes between 1 and 2 rats, the value was rounded up to 2. A color-coded version of this table is provided in Supplementary Table S1, where shading intensity reflects the required sample size (darker red for smaller values, lighter for larger). Supplementary Table S1 includes embedded macro code for dynamic cell highlighting based on value ranges.

Study Groups	ROI	β-APP	NSS	MRI				
				FA	RA	AD	MD	RD
DAI mild	Thalamus	*n* = 2	*n* = 15	*n* = 40	*n* = 47	*n* = 12,520	*n* = 236	*n* = 29
	Hypothalamus	*n* = 2		*n* = 41	*n* = 41	*n* = 691	*n* = 11,862	*n* = 529
	Neocortex	*n* = 2		*n* = 11	*n* = 8	*n* = 15	*n* = 60	*n* = 2078
	Hippocampus	*n* = 2		*n* = 49	*n* = 35	*n* = 30	*n* = 40	*n* = 218
	Corpus Callosum	*n* = 2		*n* = 16	*n* = 19	*n* = 1289	*n* = 1960	*n* = 49
	W. Brain			*n* = 6	*n* = 11	*n* = 74	*n* = 1584	*n* = 112
DAI moderate	Thalamus	*n* = 2	*n* = 6	*n* = 4	*n* = 3	*n* = 26	*n* = 279	*n* = 24
	Hypothalamus	*n* = 2		*n* = 7	*n* = 6	*n* = 1092	*n* = 342	*n* = 2074
	Neocortex	*n* = 3		*n* = 2	*n* = 2	*n* = 13	*n* = 302	*n* = 40
	Hippocampus	*n* = 3		*n* = 7	*n* = 5	*n* = 40	*n* = 201	*n* = 218
	Corpus Callosum	*n* = 2		*n* = 6	*n* = 7	*n* = 60	*n* = 2274	*n* = 28
	W. Brain	*n* = 2		*n* = 2	*n* = 2	*n* = 22	*n* = 1271	*n* = 21
DAI severe	Thalamus	*n* = 2	*n* = 5	*n* = 2	*n* = 3	*n* = 7	*n* = 12	*n* = 297
	Hypothalamus	*n* = 2		*n* = 6	*n* = 6	*n* = 19	*n* = 2	*n* = 16
	Neocortex	*n* = 3		*n* = 5	*n* = 4	*n* = 14	*n* = 86	*n* = 520
	Hippocampus	*n* = 3		*n* = 5	*n* = 3	*n* = 12	*n* = 25	*n* = 2109
	Corpus Callosum	*n* = 2		*n* = 91	*n* = 194	*n* = 14	*n* = 9	*n* = 29
	W. Brain			*n* = 2	*n* = 2	*n* = 3	*n* = 7	*n* = 1137

**Table 5:** Schematic summary comparing the sensitivity and required sample size for each assessment modality in detecting mild, moderate, and severe DAI. The table summarizes the relative sensitivity and estimated group sizes needed to detect injury using histological analysis (β-APP immunostaining), DTI metrics, and NSS. DTI metrics are grouped by commonly reported parameters: FA and RA, which showed strong correlations with histological and functional outcomes, and RD and MD, which were less sensitive and required substantially larger sample sizes.

Assessment Modality	Sensitivity (Mild)	Sensitivity (Moderate)	Sensitivity (Severe)	Typical Group Size Needed	Key Features
Histology	High	High	High	2–3	Well-established method for detecting axonal pathology (β-APP); labor-intensive; invasive
DTI (FA/RA)	Moderate	High	High	2–49+ (varies by ROI)	Strong correlations with histology and NSS; sensitive to microstructural changes
DTI (RD/MD)	Low	Low–Moderate	Moderate	7–12,000 (variable)	Less robust correlations; requires large N to detect subtle changes
NSS	Moderate	High	High	2–15	Non-invasive; correlates well with both DTI (FA/RA) and histology; functional readout

## Data Availability

Data is available upon reasonable request.

## Supplementary Materials

The following supporting information can be downloaded at: https://www.mdpi.com/article/10.3390/ijms26157333/s1.
